# Study on the Critical Wind Speed of a Resonant Cavity Piezoelectric Energy Harvester Driven by Driving Wind Pressure

**DOI:** 10.3390/mi10120842

**Published:** 2019-12-01

**Authors:** Xia Li, Zhiyuan Li, Qiang Liu, Xiaobiao Shan

**Affiliations:** 1 School of Mechanical and Power Engineering, Zhengzhou University, Zhengzhou 450001, China; ggyuan11@163.com (Z.L.); 15093367407@163.com (Q.L.); 2 State Key Laboratory of Robotics and System, Harbin Institute of Technology, Harbin 150001, China

**Keywords:** piezoelectric energy harvester, cut-in wind speed, cut-out wind speed, energy conservation method, critical stress method

## Abstract

In order to solve the problem of continuous and stable power supply for vehicle sensors, a resonant cavity piezoelectric energy harvester driven by driving wind pressure was designed. The harvester has an effective working range of wind speed. According to the energy conservation law, the cut-in (initial) wind speed of the harvester was solved. The pressure distribution law of the elastic beam in the flow field was studied by the Fluent software package, and the results were loaded into a finite element model with a method of partition loading. The relationship between the wind speed and the maximum principal stress of the piezoelectric cantilever beam was analyzed, and the critical stress method was used to study the cut-out wind speed of the energy harvester. The results show that the cut-in wind speed of the piezoelectric energy harvester is 5.29 m/s, and the cut-out wind speed is 24 m/s. Finally, an experiment on the power generation performance of the energy harvester was carried out. The experimental results show that the cut-in and cut-out wind speeds of the piezoelectric energy harvester are 5 m/s and 24 m/s, respectively, and the best matching load is 60 kΩ. The average output power, generated by the harvester when the driving wind speed is 22 m/s, is 0.145 mW, and the corresponding power density is 1.2 mW/cm^3^.

## 1. Introduction

In order to solve the environmental problems caused by automobile exhaust, some automobile companies cooperate with the government to implement the new energy “shared car” services in urban areas, with a maximum speed of no more than 70 km/h. Shared cars have more microsensors than ordinary fuel vehicles. Currently, microsensors are mainly powered by chemical batteries [1,2]. Chemical batteries have the characteristics of a large volume, low energy density, and short service life, and they also need to be replaced regularly [3,4]. These features increase the maintenance cost of shared vehicles. If the renewable energy that exists in the ambition environment can be directly converted into electricity to power microsensors, this problem can be solved very well [5,6]. The process of driving a car produces a lot of energy that can be recycled, such as heat energy [7,8], vibration energy [9,10,11] and wind energy [12]. Wind-induced vibration energy, which occurs when the car is driving, can be directly converted into electrical energy [13,14]. In recent years, there has been much research on wind-induced vibration energy recovery. There are three main types of electromechanical energy conversions, namely, piezoelectric, electromagnetic, and electrostatic [15,16]. Among them, the piezoelectric energy collecting device has the advantages of having good system compatibility, a simple structure, and a high energy density [17]. This makes piezoelectric energy harvesting a promising approach to energy recovery.

Researchers have developed many effective miniature wind-capacitor energy harvesting devices using electromagnetic or piezoelectric principles. The wind energy harvesters are mainly classified into turbine harvesters [18,19], windmill harvesters [20], reed harvesters [21,22,23,24,25,26,27], and other new structures [28,29,30,31,32,33]. Florian Herrault et al. [18] designed a microturbine electromagnetic generator. The four-pole six-turn/pole NdFeB generator exhibits up to 6.6 mW of AC electrical power across a resistive load at a rotational speed of 392,000 r/min. This milliwatt-scale power generation indicates the feasibility of such ultra-small machines for low-power applications. Sardini [19] et al. designed a turbo-type microgenerator to supply energy to the sensor that measures air temperature and speed. When the wind speed is 9 m/s, the electromagnetic generator matches the resistance of 500 Ω, and electric power with an average power of 45 mW can be obtained. Priya [20] designed a windmill-type piezoelectric energy harvester with a maximum output power of 7.5 mW when the wind speed is 10 mph and the matching resistance is 6.7 kΩ. Tan Y. K. et al. [21] designed a reed-type wind energy recovery device that uses a PZT (lead zirconate titanate) piezoelectric material to power the wind speed sensor. The wind energy harvester can start with a cut-in wind speed of 2 m/s and a cut-off wind speed of 7.5 m/s. When the wind speed is 6.7 m/s and the load is 220 kΩ, the maximum output voltage is 8.8 V, and the maximum output power is 155 μW. 

The reed-type wind energy piezoelectric energy harvester can be divided into three types according to the excitation mode, namely, the resonant cavity type [22,23,24], blunt body spoiler type [25,26], and direct excitation type [27]. D. St. Clair et al. [22] proposed a wind-pressure energy-capturing device based on self-excited airflow oscillation using a single reed and a cavity structure. When the wind speed is 7.5–12.5 m/s, the wind pressure-capture device can obtain an output power of 0.1 mW to 0.8 mW. They found that the resonant cavity-based piezoelectric energy harvester does not require any external vibration sources, thus eliminating the bandwidth problems associated with vibratory energy harvesters. Bibo Amin et al. [23] established an analytical electromechanical model to predict the response characteristics of a wind-pressure energy-capturing device based on self-excited airflow oscillation using a single reed and a cavity structure, and the effectiveness of the model was verified by experiments. In order to improve the vibration response of the wake excitation and increase the output power, L. A. Weinstein et al. [25] added a fin device at the end of the piezoelectric vibrator to make the natural frequency close to the vortex a shedding frequency. Song Rujun et al. [26] proposed a vortex-induced vibration-type piezoelectric energy-trapping device, composed of a piezoelectric cantilever beam and a terminal cylinder. They established its mathematical model and carried out experiments. The results show that this piezoelectric energy harvester can obtain the maximum energy output during vortex-induced resonance.

The wind speed of a piezoelectric energy harvester based on the turbulent vibration of the spoiler cylinder has a narrow adaptive range, and its natural frequency needs to be matched. However, the role of the spoiler cylinder is to increase the degree of chaos in the fluid flow field, and it can increase the pressure of the local fluid. It is not necessary to match the wind speed of the resonant cavity piezoelectric energy harvester. The amplitude response characteristics of the resonant cavity piezoelectric energy harvester are as follows: When the wind speed is low, the fluid kinetic energy cannot overcome the damping of the fluid flowing in the cavity, and the displacement response of the cantilever beam and voltage output is 0. When the wind speed exceeds a certain threshold, the cantilever beam produces self-sustaining and finite period oscillates, and as the wind speed increases, both the amplitude response and the voltage output increase.

In this paper, in order to achieve the purpose of continuous and stable power supply for the vehicle-mounted sensor on the shared car, the traditional resonant cavity piezoelectric energy harvester was improved: A Helmholtz resonant cavity was added to the end of the main cavity, and a spoiler cylinder was added at the entrance of the main cavity. Both the spoiler cylinder and the Helmholtz resonator increase the gas pressure in the main cavity to improve the energy capture efficiency of the harvester. The resonant cavity piezoelectric energy harvester has an effective wind speed range, in which the piezoelectric energy harvester can work effectively, otherwise the energy harvester will not work or even be destroyed. In order to discuss whether the harvester can be installed on a shared car, the effective wind speed range of this harvester has been studied. The energy conservation method was used to solve the cut-in wind speed (threshold wind speed required to start the harvester), and the critical stress method was used to solve the cut-out wind speed (maximum wind speed above which operating could result in harm to the structure) of the energy harvester. Herein, the experimental research that has been carried out is described in detail.

## 2. Structure and Working Principle

**Figure 1:**
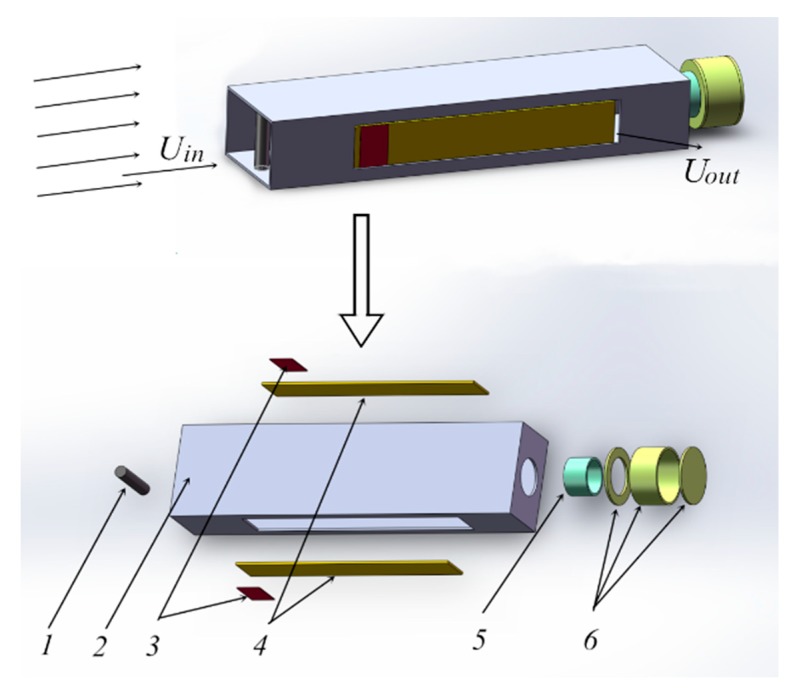
Schematic of the resonant cavity piezoelectric energy harvester: (**1**) Spoiler cylinder; (**2**) main cavity; (**3**) piezoceramics; (**4**) substrate; (**5**) Helmholtz cavity short tube; (**6**) Helmholtz resonator cavity.

This piezoelectric energy harvester was designed as a composite reed resonator structure, consisting of a structural module and two piezoelectric power generation modules, as shown in Figure 1. The structural module is composed of a main cavity, a spoiler cylinder, a Helmholtz cavity short tube, and a Helmholtz resonator. The two piezoelectric power generation modules are symmetrically distributed on the left and right sides of the main cavity, where one end of which is fixed to the main cavity body, and the other end is free to form an elastic cantilever beam structure. The spoiler cylinder is fixed at the inlet of the main cavity, and the Helmholtz cavity is fixed at the tail of the main cavity. The piezoelectric power generation module is composed of a metal substrate and piezoceramics, and the piezoceramics are attached to the root of the fixed end of the substrate.

When the car is running, the airflow enters the main cavity of the harvester from the air inlet of the main cavity (*U*_in_). The air in the main cavity is compressed, then the pressure is increased, the cantilever beam is bent and deformed, and the air outlet of the main cavity (*U*_out_) is opened. As a result, the air pressure in the main cavity is reduced, the elastic beam is reset, and the air outlet is closed. Thus, a repeating process is formed. The piezoelectric cantilever beam is periodically and repeatedly deformed to generate charges of opposite polarities in cycles on the upper and lower surfaces of the piezoelectric ceramic sheet. The piezoelectric ceramic sheets and loads form a closed energy output circuit that outputs electrical energy, thereby converting wind pressure energy into electrical energy.

## 3. Research on Cut-in Wind Speed 

The piezoelectric energy harvester has an effective wind speed range, and the minimum wind speed that enables the piezoelectric energy harvester to start capturing energy is called the cut-in wind speed, which is also called the lower critical wind speed. In this paper, the energy conservation method is used to solve the cut-in wind speed.

### 3.1. Energy Conservation Analysis of the Harvester in one Cycle

The airflow enters the piezoelectric energy harvester from the inlet of the main cavity and flows out from the outlet. In this process, the reduced wind pressure energy is partially consumed by the piezoelectric damper’s own damping force, and the other part acts on the piezoelectric harvester converted into electrical energy. Suppose there is a critical wind speed, at which the kinetic energy of the airflow is just consumed by the damping of the piezoelectric trap itself, so that the piezoelectric trap cannot capture energy. This critical velocity of the airflow entering the cavity is the cut-in wind speed.

The damping of the piezoelectric energy harvester consists of three parts, namely, the damping of the material used in the piezoelectric energy harvester (the phenomenon of mechanical energy loss caused by the friction among the internal grains of the material caused by the action of the trap in the wind field), the fluid damping generated by the various structures of the piezoelectric energy harvester after the fluid enters it, and piezoelectric damping produced by piezoelectric ceramic when piezoelectric cantilever beam vibrates. The material of piezoelectric energy harvester produces little damping and can be neglected during the research process. When the wind speed acting on the piezoelectric cantilever beam is equal to the cut-in wind speed, the piezoelectric cantilever beam is not deformed, and the piezoelectric ceramic piece does not generate electric energy. As such, there is no piezoelectric damping. 

The cut-in wind speed can be obtained by the energy conservation method. When the energy conservation method is used to calculate the starting wind speed, the work done by the fluid damping force generated by each part of the structure is equal to the kinetic energy of the fluid entering the cavity. The damping of the piezoelectric energy harvester mainly includes the fluid damping force generated by the turbulent cylinder and the fluid damping force generated by the inner surface of the cavity.

The fluid kinetic energy in one cycle is calculated by Equation (1):

(1) $$E_{p}=\frac{1}{2}mv^{2}=\frac{1}{2}\rho \left(A_{0}\cdot vT\right)v^{2}=\frac{1}{2}\rho A_{0}v^{3}T$$

where *E_p_* is defined as the kinetic energy of the fluid in a cycle, *ρ* is defined as the density of the air, *A*_0_ is defined as the inlet area of the main cavity, *v* is defined as the velocity of the fluid at the entrance of the harvester, and *T* is defined as the vibration period of the piezoelectric cantilever.

The work done by the piezoelectric energy harvester structure damping in one cycle is calculated by Equation (2):

(2) $$W=W_{d}+W_{q}$$

where *W* is defined as the work done by the structural damping force in a cycle, *W_d_* is defined as the work done by the fluid damping force generated by the spoiler cylinder in a cycle, *W_q_* is defined as the work done by the fluid damping force generated on the inner surface of the main cavity and the Helmholtz resonator.

### 3.2. Work Done by the Spoiler Damping Force 

#### 3.2.1. Theoretical Analysis

The average resistance coefficient of the cylinder unit length is [34]:

(3) $$C_{d}=\frac{F_{d}}{\frac{1}{2}\rho Dv^{2}}$$

The fluid damping force generated by the spoiler cylinder of length *L* is:

(4) $$F_{d}=\frac{1}{2}\rho C_{d}DLv^{2}$$

where *C_d_* is defined as the resistance coefficient of the spoiler cylinder (*C_d_* is 1.11, according to the empirical value [34]), and *D* and *L* are respectively defined as the diameter and length of the spoiler cylinder.

The work of the damping force generated by the spoiler cylinder in a cycle is given by Equation (5):

(5) $$W_{d}=F_{d}\cdot x=F_{d}\cdot vT=\frac{1}{2}\rho C_{d}DLv^{3}T$$

where *x* represents the displacement of the spoiler cylinder relative to the fluid for a cycle.

#### 3.2.2. Simulation Verification of the Value of Resistance Coefficient

The resistance coefficient is a function of the Reynolds number [34]. The Reynolds number is related to the velocity, so the resistance coefficient is related to the speed. 

In order to verify the accuracy of the resistance coefficient selected according to the empirical value when solving the starting wind speed, the resistance coefficient of the spoiler cylinder is calculated by the Fluent software package, then compared with the empirical value resistance coefficient selected. If the error is within ±5%, it shows that the resistance coefficient obtained by the empirical value is reasonable when the energy conservation method is used to calculate the wind speed of the resonant cavity piezoelectric harvester. 

**Figure 2:**
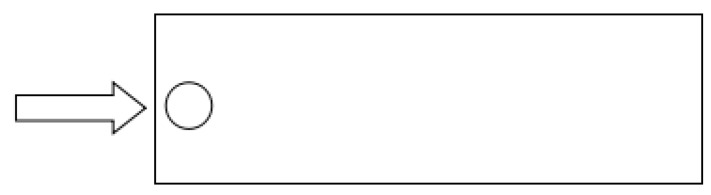
Simplified flow field model of the main cavity.

In order to facilitate the study, this paper neglects the influence of the length of the cylinder. By simplifying the three-dimensional model of the main cavity into two-dimensional model, the influence of the disturbing cylinder on the model is studied. The simplified model is shown in Figure 2.

The Kármán vortex street effect occurs when the fluid passes through a cylinder. At this time, the shedding frequency of the fluid vortex in the flow channel [35] is:

(6) $$f=S_{t}\frac{v}{\varepsilon D}$$

where

(7) $$\varepsilon =1-\frac{2}{\pi }\left[\frac{D}{\Phi }\sqrt{1-{\left(\frac{D}{\Phi }\right)}^{2}}+\arcsin\frac{D}{\Phi }\right]$$

where *S_t_* is the Strouhal number, which indicates the relative relationship between the fluid kinetic energy and the inherent energy of the system. When the fluid flows over the spoiler cylinder, according to the calculation formula of Reynolds number, $Re=\frac{\rho vD}{\mu }$ (*v* takes the values of 1–30 m/s; *ρ* is air density; *μ* is hydrodynamic viscosity; *D* is the diameter of the spoiler cylinder), where the range of *Re* is from 336.7 to 10,101.2. It is common practice to assume *S_t_* = 0.21 in the sub-critical range, 300 < *Re* < 1.5 × 10^5^ [36]; *ε* represents the ratio of the area of the arcuate faces on both sides of the cylinder to the cross-sectional area of the intake passage; Φ represents the hydraulic diameter of the inlet.

When the flow field dynamic characteristics of the spoiled cylinder are analyzed, the boundary conditions are as follows.

1. The flow field calculation domain is based on Navier–Stokes equations (N-S equation) and is simulated using the realizable *k*-*ε* turbulence model. The flowing medium is ideally air at 20 °C, with a density of 1.205 kg/m^3^, and a dynamic viscosity coefficient of 1.7894 × 10^−5^ Pa·s.
2. The inlet and outlet of the model are respectively set as a velocity inlet and a pressure outlet, and the absolute pressure at the outlet is 1.01 × 10^5^ Pa. The wall is set to a fixed wall with no slip and an ambient temperature of 20 °C.
3. In order to accurately obtain the dynamic characteristics of the fluid in the flow field, the basic unit size of the flow field is set to 2 mm, and the meshes of the spoiler cylinder and the two side walls are refined. The number of nodes and grids are 38,383 and 73,990 respectively, and the average mesh quality is 0.97.
4. In order to refine the transient dynamics analysis results of the Kármán vortex generated by the fluid passing through the spoiler cylinder, the time step in the flow field is set to 1/10 of the detuning period of the spoiler cylinder.

**Figure 3:**
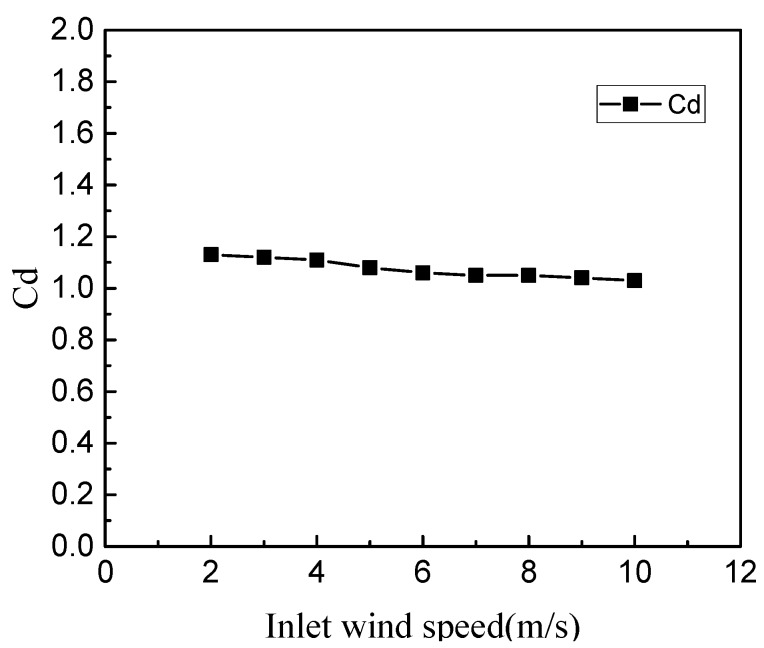
Relationship between inlet wind speed and resistance coefficient.

The inlet wind speed is set from 2 m/s to 10 m/s, and the step length is 1 m/s. The remaining parameters are not changed. Finally, the resistance coefficient of the spoiler can be calculated. The results from our analysis are shown in Figure 3.

It can be seen from Figure 3 that when the inlet wind speed is different, the resistance coefficient also changes, but the change is not obvious. Therefore, the influence of wind speed on the resistance coefficient of the turbulent cylinder can be ignored. The average value of the coefficient of resistance of the turbulent cylinder is 1.07. The error is 3.6% when compared with the empirical value of 1.11, and is less than 5%, which is within the allowable range. Therefore, it can be verified that the empirical value of the resistance coefficient selected by the empirical value is reasonable when the energy method is used to solve the cut-in wind speed.

### 3.3. Work Done by the Damping Force Generated by the Main Cavity and the Inner Surface of the Helmholtz Resonator

The friction of the flat wall surface per unit area [34] is calculated by Equation (8):

(8) $$\tau_{f}=0.3232\sqrt{\frac{\mu \rho v^{3}}{x}}$$

where *μ* represents the air viscosity coefficient.

The fluid damping force produced on the upper and lower surfaces of the inner wall of the main cavity is calculated by Equation (9) [34]:

(9) $$F_{f}=2\left(\int_{0}^{L_{B}}\tau_{f}\cdot B\mathrm{dx}-2\int_{0}^{2R}\tau_{f}\cdot \sqrt{R^{2}-{\left(R-x\right)}^{2}}\mathrm{dx}\right)$$

Equation (8) can be substituted for Equation (9) and then simplified:

(10) $$F_{f}=1.2928\sqrt{\mu \rho v^{3}}(\sqrt{L_{B}}\cdot B-\frac{2}{3}\left(2R{)}^{\frac{3}{2}}\right)$$

where *L_B_* and *B* denote the length and width of the main cavity, respectively, and *R* denotes the radius of the spoiler cylinder (2*R* = *D*).

Similarly, the fluid damping force generated on the left and right surfaces of the inner wall of the main cavity is given by Equation (11) [34]:

(11) $$F_{h}=2\left(\int_{0}^{L_{B}}\tau_{f}\cdot h\mathrm{dx}-2\int_{L_{2}}^{L_{3}}\tau_{f}\cdot \Delta h\mathrm{dx}-\int_{L_{3}}^{L_{3}+\Delta b}\tau_{f}\cdot b\mathrm{dx}\right)$$

where *h* denotes the height of the main cavity; *b* denotes the width of the substrate; ∆*h* denotes the gap between the main cavity and the substrate in the width direction; ∆*b* denotes the gap between the main cavity and the substrate in the length direction; *L*_2_ and *L*_3_ denote the distance from the entrance of the main cavity to the front end and the end of the substrate, respectively.

Then, the fluid damping generated on the inner wall surface of the main cavity can be expressed by Equation (12):

(12) $$F_{h}=1.2928\sqrt{\mu \rho v^{3}}\left(\sqrt{L_{B}}\cdot h-2\left(\sqrt{L_{2}}-\sqrt{L_{3}}\right)\cdot \Delta h-\left(\sqrt{L_{3}+\Delta b}-\sqrt{L_{3}}\right)\cdot b\right)$$

Similarly, the fluid damping generated on the inner surface of the Helmholtz resonator at the end of piezoelectric energy harvester can be calculated by Equation (13):

(13) $$F_{m}=1.2928\pi \sqrt{\mu \rho v^{3}}\left(\left(\sqrt{L_{4}}-\sqrt{L_{B}}\right)\cdot R_{1}+\left(\sqrt{L_{5}}-\sqrt{L_{4}}\right)\cdot R_{2}\right)$$

where *L*_4_ is defined as the distance from the entrance of the main cavity to the end of the short tube, *L*_5_ is defined as the distance from the entrance of the main cavity to the end of the Helmholtz resonator cavity, *R*_1_ is defined as the radius of the short tube, and *R*_2_ is defined as the radius of the Helmholtz cavity.

### 3.4. Numerical Calculation of Cut-in Wind Speed

The work done by the fluid damping force of the main cavity in one cycle can be calculated by Equation (14):

(14) $$W_{q}=\left(F_{f}+F_{h}+F_{m}\right)\cdot x=\left(F_{f}+F_{h}+F_{m}\right)\cdot vT$$

According to the law of conservation of energy:

(15) $$E_{p}=W=W_{d}+W_{q}$$

Substituting Equations (1), (5), and (14) into Equation (15), Equation (15) can be converted into Equation (16):

(16) $$\frac{1}{2}\rho A_{0}v^{3}T=F_{d}\cdot vT+\left(F_{f}+F_{h}+F_{m}\right)\cdot vT$$

Substituting Equations (4), (10), (12), and (13) into Equation (16), the cut-in wind speed can be obtained by solving Equation (17).

(17) $$\rho C_{d}DL+2.5856\sqrt{\mu \rho v}\left[\begin{array}{l} (\sqrt{L_{B}}\cdot B-\frac{2}{3}\left(2R{)}^{\frac{3}{2}}\right)+(\sqrt{L_{B}}\cdot h-2\left(\sqrt{L_{3}-L_{2}}\right)\cdot \Delta h-(\sqrt{L_{3}+\Delta b}\\ -\sqrt{L_{3}})\cdot b)+\pi \left(\sqrt{L_{4}}-\sqrt{L_{B}}\right)R_{1}+\pi \left(\sqrt{L_{5}}-\sqrt{L_{4}}\right)R_{2} \end{array}\right]-\rho A_{0}=0$$

The values of the parameters in Equation (17) are shown in Table 1.

Equation (17) can be solved by mathematics software, and the cut-in wind speed of the piezoelectric energy harvester obtained here was 5.29 m/s.

## 4. Solution of Wind Cut-off Velocity by Stress Critical Value Method

When the airflow enters the cavity of the piezoelectric harvester, the piezoelectric cantilever beam arranged on both sides of the cavity will bend and deform. When the piezoelectric cantilever beam can work normally and is not damaged, the maximum airflow speed that it can withstand is called the cut-out wind speed, also called the upper critical wind speed. If the inlet wind speed is higher than this critical wind speed, the cantilever beam or the piezoelectric ceramic piece may break and cause a failure. On the contrary, if the airflow velocity is lower than the cut-off wind speed, the piezoelectric cantilever beam will not be destroyed. Therefore, the cut-out wind speed can be obtained by solving the wind speed at the point of critical stress of the piezoelectric beam.

The piezoelectric module is composed of a substrate and a piezoelectric ceramic piece, both of which must be able to work normally within the allowable range of allowable stress. The substrate material used was beryllium bronze, whose strength is 1035 MPa [37]. The piezoelectric ceramic sheet was PZT-5H, where the tensile strength under the d_31_ working mode is 75.845 MPa [38]. Taking a safety factor of 1.3 [39], the allowable stresses for the substrate and the piezoelectric ceramic piece are 796 MPa and 58.345 MPa, respectively. Therefore, it is necessary to ensure that the maximum stress of the substrate and the piezoelectric piece when the piezoelectric cantilever is in operation does not exceed their respective allowable stresses.

The Fluent software package was used to analyze the pressure distribution on the elastic beam of the piezoelectric trap when the airflow acted on the elastic beam in the flow field. 

The specific process of fluid simulation was as follows:

1. Model import and establishment of the flow field: The resonant cavity piezoelectric energy harvester structure model was modeled by SolidWorks and saved in the IGS format and then imported into Fluent. The flow field was established according to the size parameters of the energy harvester. Generally, the flow field size was about 5 to 10 times that of the research object. The length, width, and height of the flow field established in this paper was 1000 mm, 400 mm, and 200 mm, respectively. Finally, the fluid inlet and the fluid outlet were set.
2. Meshing: In this paper, an unstructured tetrahedral mesh was used to divide the flow field, and the piezoelectric trap was partially refined by a local mesh. The number of nodes was 187,126, the number of elements was 1,049,576, and the average mesh quality was 0.904.
3. Physical model selection and boundary condition setting: According to the calculation formula of Re, the Re values at different flow rates were respectively calculated. When Re < 2300, the physical model was set to laminar flow, and when *Re* > 2300, it was set to turbulent flow. The most commonly used turbulence model is the standard *k*-*ε* model. The standard *k*-*ε* model introduces the fluid kinetic energy equation (*k*) and the loss rate equation (*ε*). Finally, the solutions of *k* and *ε* were obtained, and the values of *k* and *ε* were used. The fluid viscosity was calculated and the solution of Reynolds stress was obtained by the Boussinesq hypothesis. However, the standard *k*-*ε* model has a large error in solving the swirl problem. The RNG *k*-*ε* model is an improved model of the Realizable *k*-*ε* model. In this paper, the improved RNG *k*-*ε* model was selected for numerical simulation.
  Boundary condition setting: The inlet wind speed was 15 m/s, outlet pressure was the standard atmospheric pressure, and wall surface was set to no slip. The monitoring was set to monitor the pressure value of the cantilever beam on both sides of the piezoelectric energy harvester.
4. Initialization calculation and postprocessing: Global initialization was required before starting the calculation. The initialization method for this article was standard initialization. The initial pressure was set to the standard atmospheric pressure. Here, the postprocessing mainly extracts and collates the pressure data of the cantilever beams on both sides.

**Figure 4:**
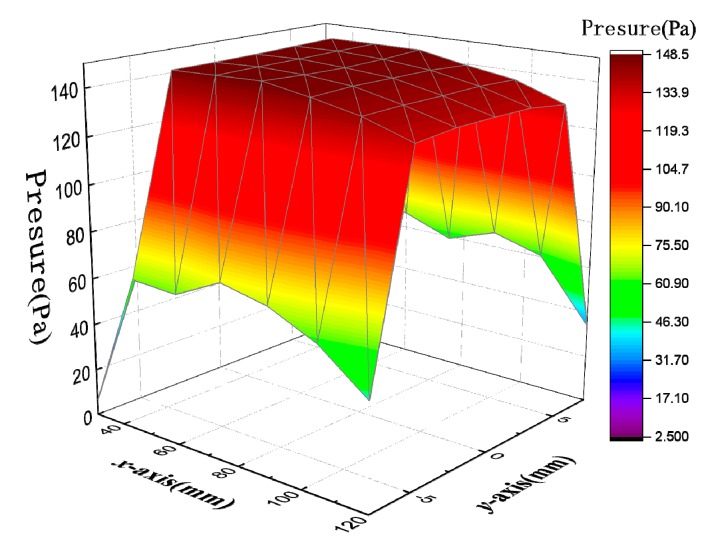
Distribution of the pressure on the substrate in the wind field.

The pressure distribution of each node on the inner surface of the substrate in the wind field was extracted from the analysis results. The pressure distribution law is shown in Figure 4, where the *x*-axis is the length of the substrate, with a range of 30–120 mm, the *y*-axis is the width of the substrate, with a range from −7.5 mm to 7.5 mm, and the *z*-axis is the pressure value, with a range from 0 Pa to 150 Pa.

**Figure 5:**
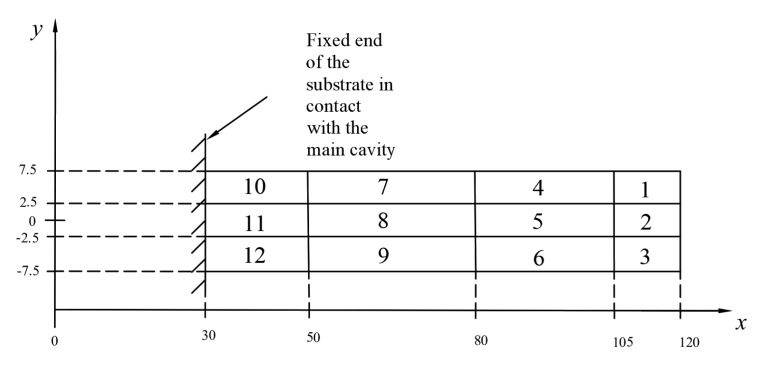
Division of substrates.

As can be seen from Figure 4, the pressure in the x-direction was increased first and then decreased, but the change was small. When the y-coordinate range was from −7.5 mm to −2.5 mm, the pressure increased linearly with the increase of the y-coordinate value. When the y-coordinate range was from −2.5 mm to 2.5 mm, the pressure did not obviously change with the increase of the y-coordinate value. When the y-coordinate range was from 2.5 mm to 7.5 mm, the pressure decreased linearly with the increase of the y-coordinate value. According to the distribution of wind pressure on the substrate in the wind field, the substrate can be divided into 12 regions, as shown in Figure 5.

As shown in Figure 5, the pressure values of regions 4, 5, 6, 7, 8, and 9 are not much different, and approximate a rectangular area of equal value. The four regions of regions 1, 3, 10, and 12 are approximately triangular. Regions 2 and 11 are approximately rectangular. For the approximate isosurface area, a plurality of values may be averaged, and the triangular region can take the corresponding pressure value at the median line as the average value of the region, and the rectangular region takes the pressure value at the intermediate symmetry axis as the average value of the region.

The pressure of each region at different wind speeds is obtained by changing the inlet wind speed. The static analysis of the piezoelectric cantilever beam was performed using the Ansys software package according to the above loading method. The specific simulation details are described below:

1. Define element type and material parameters: In this paper, the piezoelectric coupling analysis of piezoelectric cantilever beam in piezoelectric energy harvester was carried out by ANSYS 14.5. The piezoelectric ceramic sheet element type was the coupling element Solid226, and the cantilever beam element was the structural element Solid45.
2. Meshing: In this paper, the cantilever beam was meshed by the means of mapping grid method. We used the glue command to combine the nodes on the contact surface of the piezoelectric piece with the substrate. The grid element size of the piezoelectric ceramic sheet, the bonded portion of the substrate, and the piezoelectric ceramic sheet were all set to 0.2 mm. The grid element size of the substrate root was 0.3 mm, and the grid element size of the other portion of the substrate was 0.6 mm.
3. Boundary conditions: A fixed end constraint was added to the left end of the piezoelectric cantilever beam. The upper surface node of the piezoelectric ceramic sheet was coupled to the upper electrode. The lower surface node of the piezoelectric ceramic sheet was coupled to the lower electrode, and the coupling voltage was set to 0 V.
4. Apply load and solve: The pressure was applied to different regions of the piezoelectric sheet by the method of partition loading, described above, and then the solution was calculated. After the calculation was completed, the Mises stress results on the piezoelectric beam were extracted.

**Figure 6:**
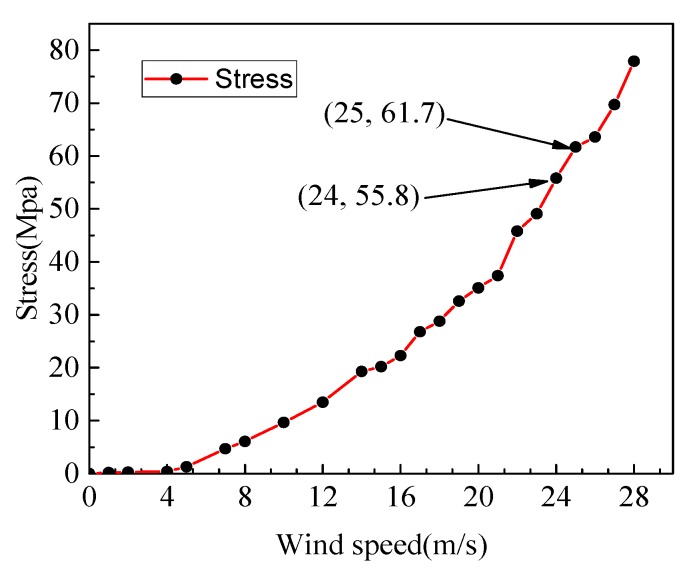
Relationship between wind speed and Mises stress of piezoelectric piece.

After the simulation calculation, since the maximum Mises stress generated on the substrate did not exceed 200 MPa, which is much smaller than its allowable stress, the relationship between the maximum Mises stress on the substrate and the wind speed is not listed here. Figure 6 is a graph showing the relationship between the maximum Mises stress of the piezoelectric piece and the wind speed.

It can be seen from Figure 6 that the maximum Mises stress value of the piezoelectric piece increases with the increase of the wind speed. When the wind speed is lower than 5 m/s, the maximum Mises stress value of the piezoelectric piece approaches 0. When the wind speed is greater than 25 m/s, the maximum Mises stress of the piezoelectric piece exceeds 61.7 MPa, which is greater than the allowable stress of the piezoelectric piece. When the wind speed is between 24 m/s and 25 m/s, there is a wind speed value such that the maximum Mises stress of the piezoelectric piece is exactly equal to the allowable stress of the piezoelectric piece. In order to ensure the safety of the piezoelectric cantilever beam structure, the cut-out wind speed of the piezoelectric energy harvester was determined to be 24 m/s. This speed is greater than the maximum speed that a shared car travels in the city, so the piezoelectric energy harvester installed on the shared car can work properly.

## 5. Experimental Study

**Figure 7:**
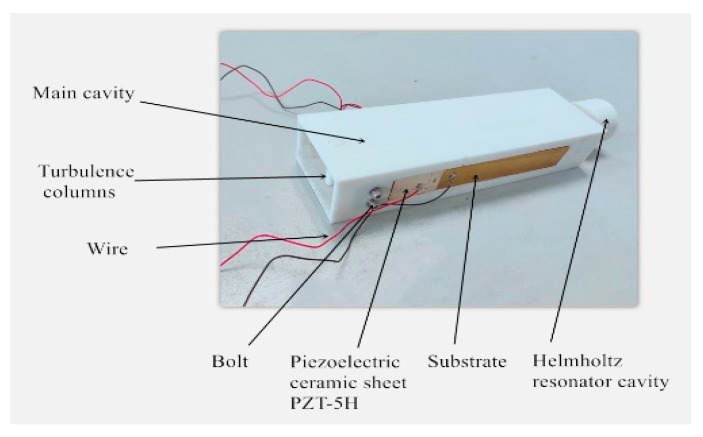
Resonant cavity piezoelectric energy harvester prototype.

In order to determine the output characteristics of the resonant cavity piezoelectric energy harvester and verify the correctness of the critical wind speed calculation results, a prototype of the resonant cavity piezoelectric energy harvester was fabricated, as shown in Figure 7. The thickness of the piezoelectric piece and substrate were both 0.5 mm, and the remaining dimensions were the same as in Table 1.

The resonant cavity piezoelectric energy harvester model is mainly composed of wires, a main cavity, a spoiler cylinder, a bolt, two piezoelectric ceramic pieces, two substrates, and a Helmholtz resonant cavity. The main cavity, the spoiler cylinder, and the Helmholtz resonator were modeled in SolidWorks and then processed separately using 3D printing technology, where the material used was PLA. The left and right sides of the main cavity both have a rectangular groove, with a length of 96 mm and a width of 16 mm, for fixing the piezoelectric cantilever beams. The material of the substrates used was beryllium bronze, which was cut by a cutter. The piezoelectric ceramic pieces used the PZT-5H piezoelectric ceramic pieces produced by the Hebei Hongsheng Acoustic Electronic Equipment Co., Ltd. The size of the piezoelectric piece was 10 mm × 15 mm × 0.4 mm. A through-hole was formed at the entrance of the main cavity and was assembled with the spoiler cylinder. The main cavity has a round hole at the end was assembled with the Helmholtz cavity. The piezoelectric cantilever beam was fixed on both sides of the main cavity by M3 bolts. The piezoelectric cantilever beam was made by bonding the PZT-5H to the substrate by epoxy resin containing copper powder. Two wires were spot-welded on the positive surface of PZT-5H and the substrate.

**Figure 8:**
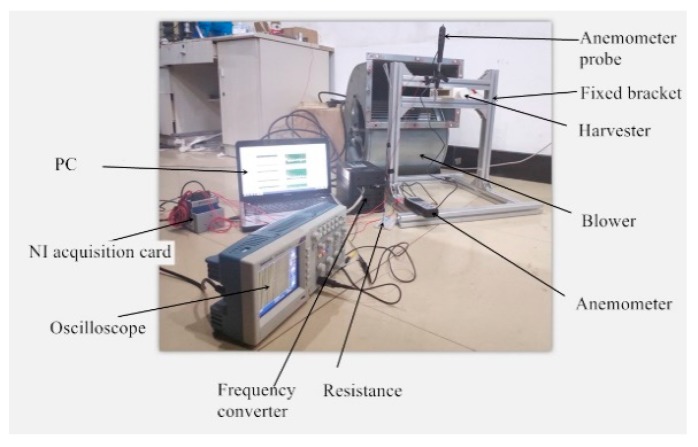
Resonant cavity piezoelectric energy harvester experimental platform.

The experiment platform, as shown in Figure 8, was constructed. The experimental study on the output characteristics of the resonant cavity piezoelectric energy harvester was carried out.

The equipment used in the experiment were as follows: A blower (LKW315L-4, Shandong Qineng Ventilator Co., Ltd., Zibo, China), with the maximum adjustment frequency of 50 Hz and a maximum speed of 1350 r/min, a NI acquisition card (NI-cDAQ-9174, National Instruments, Austin, TX, USA), an oscilloscope (ADS 1102CAL, Guorui Antai Technology Co., Ltd., Nanjing, China), a frequency converter (VFD 110B 43A Shanghai Xinyu Automation Equipment Co., Ltd., Shanghai, China), an anemometer (CTV100, Beijing Oubai Keyi Instrument Co., Ltd. Beijing, China), with a wind speed measurement range of 0–30 m/s and a sensitivity of 0.1 m/s, and a PC.

**Figure 9:**
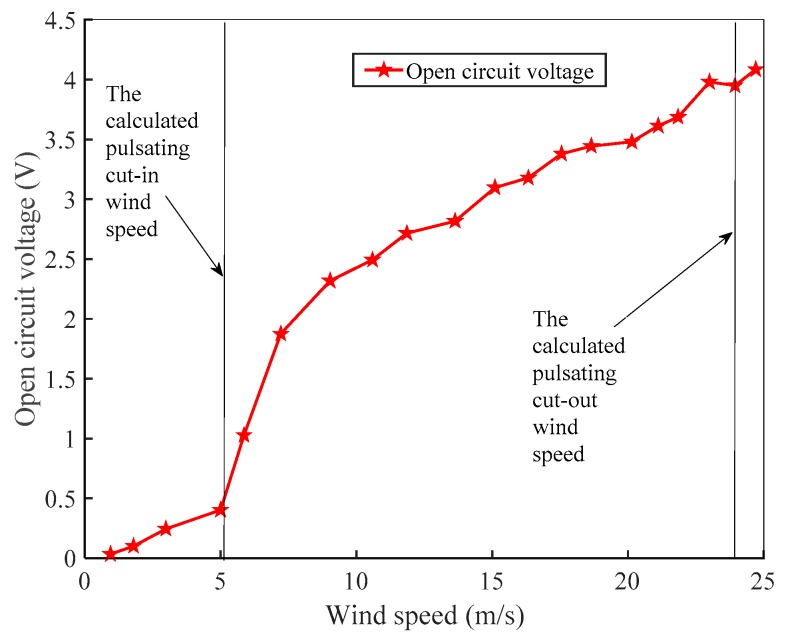
Curve of open circuit voltage with wind speed.

Since two piezoelectric cantilever beams were symmetrically mounted on both sides of the piezoelectric energy harvester, only the vibration of one side piezoelectric cantilever beam was studied. Figure 9 shows the average open circuit voltage of the piezoelectric energy harvester at different wind speeds.

As can be seen from Figure 9, the open circuit voltage of the piezoelectric harvester increases nonlinearly with the increase of the wind speed. When the wind speed increases from 0 m/s to 5 m/s, the open circuit voltage increases slowly. When the wind speed is greater than 5 m/s, the open circuit voltage increases rapidly. In the actual experiment, the external small vibration can also cause the vibration of the energy absorbing device. Considering the influence of the actual factors, the cut-in wind speed of the piezoelectric energy harvester is 5 m/s. The blower can provide a maximum wind speed of 25 m/s, and the trap can still operate at this wind speed. However, in the experiment, it was found that after the harvester worked at a large wind speed for a long time, the piezoelectric cantilever beam would undergo a slight bending deformation. Therefore, the cut-out wind speed of the piezoelectric energy harvester should be 24 m/s. The effective wind speed range of the harvester is 1.58 times that of the micropiezoelectric wind energy harvester with a resonant cavity (with an effective wind speed range of 4–16 m/s), which has a cavity size of 64 mm × 22 mm × 14 mm, with a piezoelectric beam length of 8 mm and a flexible beam length of 30 mm, as described by Du Zhigang [24].

**Figure 10:**
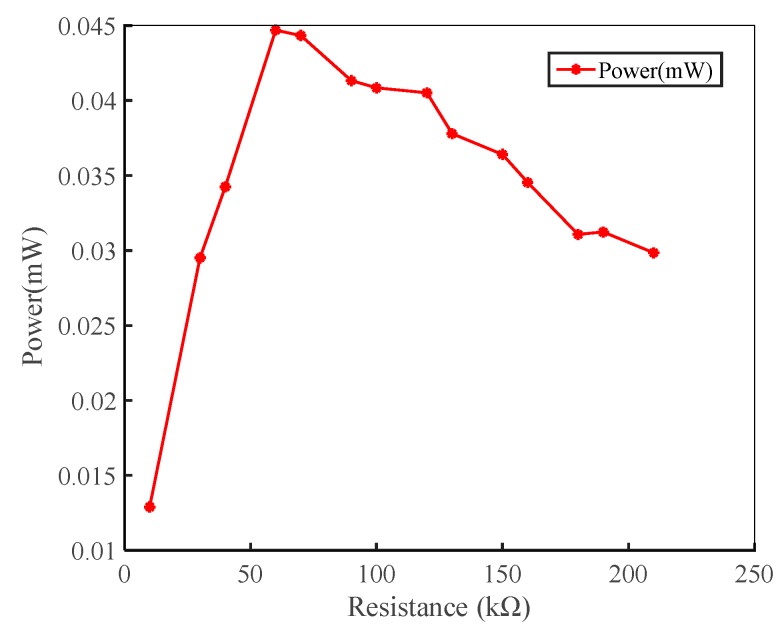
Relationship between output power and load resistance at the wind speed of 10 m/s.

The power generation performance of a piezoelectric energy harvester needs to be measured by a load resistor [40,41]. Figure 10 is a plot of output power as a function of load resistance at the wind speed of 10 m/s.

It can be seen from Figure 10 that when the wind speed is 10 m/s, the output power firstly increases and then decreases with the increase of the load resistance. When the load resistance is 60 kΩ, the output power of one side piezoelectric cantilever beam reaches a maximum of 45 μW. In this experiment, the optimal resistance of the piezoelectric energy harvester was 60 kΩ.

**Figure 11:**
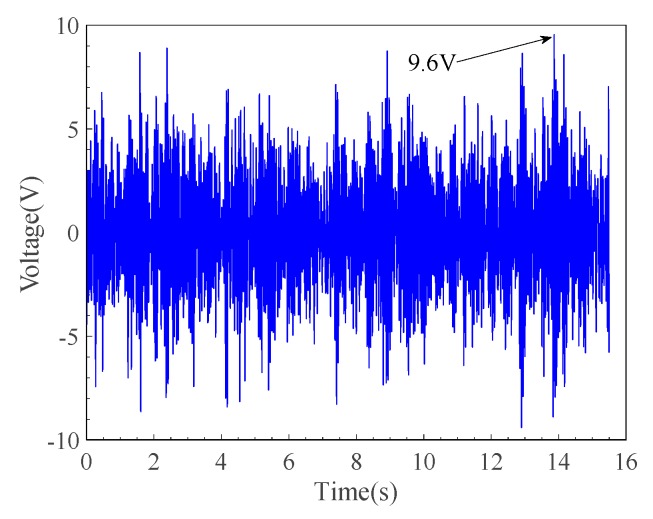
Variation of output voltage at a wind speed of 22 m/s.

Figure 11 shows the relationship of the output voltage with time at a wind speed of 22 m/s when the resistance was 60 kΩ.

It can be seen from Figure 11 that the peak output voltage of the single-sided piezoelectric cantilever beam of the piezoelectric energy harvester can reach 9.6 V when the wind speed is 22 m/s, and the peak output power obtainable at this time is 1.536 mW. The average voltage of one side of the piezoelectric cantilever beam was 2.0877 V, and the corresponding total power and power density here were 0.145 mW and 1.2 mW/cm^3^, respectively.

## 6. Conclusions

In this paper, a wind-pressure resonant cavity piezoelectric energy harvester was designed, and the working wind speed range was studied. Experimental analysis of the harvester was carried out and the following conclusions were obtained:

1. By calculating the work done by the damping generated by the various parts of the harvester in the flow field, which was set to be equal to the initial kinetic energy of the fluid, the pulsating wind speed was obtained. The resistance coefficient of the spoiler cylinder was simulated by the Fluent software package. The results show that the inlet wind speed has little effect on the resistance coefficient. The average simulation result was compared with the empirical value, and the error was within the allowable range, verifying the correctness of the resistance coefficient selected by the empirical value. The resulting cut-in wind speed of the harvester was 5.29 m/s.
2. By solving the wind speed when the piezoelectric modules are not damaged, the cut-out wind speed of the harvester was obtained. The pressure distribution of the piezoelectric cantilever beam in the flow field was obtained by Fluent software simulation. The finite element model of the piezoelectric cantilever beam was loaded by the partition loading pressure method. The relationship between the wind speed and the maximum principal stress of the piezoelectric cantilever beam was studied. The cut-out wind speed of the harvester was 24 m/s.
3. Through experimental research, it was concluded that the cut-in wind speed of the energy harvester was 5 m/s and the cut-out wind speed was 24 m/s. The best external load resistance of the harvester was 60 kΩ. The average voltage of one side piezoelectric cantilever beam was 2.0877 V, and the corresponding total power and power density were 0.145 mW and 1.2m W/cm^3^, respectively, at a wind speed of 22 m/s and an external resistance of 60 kΩ.

## Figures and Tables

**Table 1:** Initial values of each parameter.

Parameters	Values	Parameters	Values
*μ*/Pa·s	1.78 × 10^−5^	Δ*h*/mm	0.5
*ρ*/kg/m^3^	1.225	Δ*b*/mm	6
*D*/mm	5	*L*_B_/mm	150
*B*/mm	38	*L*_2_/mm	30
*R*_1_/mm	6	*L*_3_/mm	120
*R*_2_/mm	15	*L*_4_/mm	160
*h*/mm	23	*L*_5_/mm	175
*b*/mm	15	-	-
